# Rule-based deduplication of article records from bibliographic databases

**DOI:** 10.1093/database/bat086

**Published:** 2014-01-16

**Authors:** Yu Jiang, Can Lin, Weiyi Meng, Clement Yu, Aaron M. Cohen, Neil R. Smalheiser

**Affiliations:** ^1^Department of Computer Science, Binghamton University, Binghamton, NY 13902, USA, ^2^Department of Computer Science, University of Illinois at Chicago, Chicago, IL 60612, USA, ^3^Department of Medical Informatics and Clinical Epidemiology, Oregon Health & Science University, Portland, OR 97239, USA and ^4^Department of Psychiatry and Psychiatric Institute, University of Illinois at Chicago, Chicago, IL 60612, USA

## Abstract

We recently designed and deployed a metasearch engine, Metta, that sends queries and retrieves search results from five leading biomedical databases: PubMed, EMBASE, CINAHL, PsycINFO and the Cochrane Central Register of Controlled Trials. Because many articles are indexed in more than one of these databases, it is desirable to deduplicate the retrieved article records. This is not a trivial problem because data fields contain a lot of missing and erroneous entries, and because certain types of information are recorded differently (and inconsistently) in the different databases. The present report describes our rule-based method for deduplicating article records across databases and includes an open-source script module that can be deployed freely. Metta was designed to satisfy the particular needs of people who are writing systematic reviews in evidence-based medicine. These users want the highest possible recall in retrieval, so it is important to err on the side of not deduplicating any records that refer to distinct articles, and it is important to perform deduplication online in real time. Our deduplication module is designed with these constraints in mind. Articles that share the same publication year are compared sequentially on parameters including PubMed ID number, digital object identifier, journal name, article title and author list, using text approximation techniques. In a review of Metta searches carried out by public users, we found that the deduplication module was more effective at identifying duplicates than EndNote without making any erroneous assignments.

## Introduction

Record linkage and deduplication refer to the process of recognizing different items that refer to the same underlying entity, either within a single database or across a set of databases. This is a basic step in data mining that has attracted a large body of research, with most methods pursuing either statistical or rule-based approaches (1–11). A specific type of deduplication involves merging records from bibliographic databases that refer to the same article or book (this may or may not involve segmentation of free text into structured record fields) (3, 12). Due to the prevalent need for deduplication in the text mining process, several open-source tools for biomedical and generic record linkage exist [e.g. Febrl (http://datamining.anu.edu.au/linkage.html) and FRIL (http://fril.sourceforge.net/)] (12, 13). However, these require offline training and are not suitable for online deduplication of diverse types of search outputs.

We recently designed and deployed a metasearch engine, Metta, that sends and retrieves queries from five leading biomedical databases: PubMed, EMBASE, CINAHL, PsycINFO and the Cochrane Central Register of Controlled Trials (http://mengs1.cs.binghamton.edu/metta/search.action; (14)). These databases are partially overlapping but distinct in scope. PubMed focuses primarily on basic and clinical medical research, EMBASE contains a wider collection of chemistry and zoology journals, CINAHL emphasizes allied health fields (such as nursing and physical therapy), PsycINFO covers psychology and social sciences and the Cochrane Central Register contains many clinical trial articles that were presented as conference proceedings. Retrieved articles are offered to the user in either BibTeX format (abbreviated record, Table 1) or XML format (full bibliographic record). Because many articles are indexed in more than one of these databases, it is desirable to deduplicate the retrieved article records. Metta was designed in particular to satisfy the particular needs of people who are writing systematic reviews in evidence-based medicine—these users want the highest possible recall in retrieval, so it is important to err on the side of not deduplicating any records that refer to distinct articles, and it is also important to perform deduplication online in real time. We have designed our own rule-based deduplication module with these constraints in mind.

**Table 1.** Articles in BibTeX format

@article{PUBMED18812194,
Author=“Smalheiser, NR. and Lugli, G. and Torvik, VI. and Mise, N. and Ikeda, R. and Abe, K.”,
Title=“{Natural antisense transcripts are co-expressed with sense mRNAs in synaptoneurosomes of adult mouse forebrain.}”,
Journal=“Neuroscience research”,
Year=“2008”,
Volume=“62”,
Number=“4”,
Pages=“236–9”

@article{EMBASE:2008527667,
Author=“Smalheiser N.R. and Lugli G. and Torvik V.I. and Mise N. and Ikeda R. and Abe K.”,
Title=“{Natural antisense transcripts are co-expressed with sense mRNAs in synaptoneurosomes of adult mouse forebrain}”,
Journal=“Neuroscience Research”,
Year=“2008”,
Volume=“62”,
Number=“4”,
Pages=“236–239”

Shown are the PubMed and EMBASE records in BibTeX format for one typical article. Even though there are no missing or erroneous data, note variations in the author list, journal and page numbering.

The contributions of this article are as follows: we present an efficient multi-step rule-based deduplication algorithm for online deduplication. We experimentally evaluate the proposed algorithm and show that it has very good performance for high recall and online deduplication needs. We compare it with the deduplication module of a popular reference management system (EndNote) and show that our algorithm performs better. Furthermore, this algorithm has been deployed to a real metasearch system, Metta. We also make available the Java code of our algorithm and the accompanying documentation to others.

## Methods

As shown in Table 1, different databases display the same bibliographic record in different ways. They may write the author names differently (e.g. sometimes first and last names are reversed, sometimes author order is not preserved, sometimes not all authors are listed or sometimes middle initials are omitted). They may abbreviate journal names in different ways, and page numbers may be written in various ways. One must also keep in mind that some articles have no listed authors at all, and that titles may not be unique (e.g. ‘Editorial’). Errors and missing data are surprisingly common, especially in EMBASE (15). Utilizing the full records in XML format is helpful; for example ISSN numbers provide unique journal identifiers, but may not be present in all cases.

As we do not have any single ‘ID’ field with full applicability, single selectivity and full accuracy (Table 2), we are unable to determine whether two records are identical or not in one step. Thus our algorithm should consider multiple attributes, together or step by step, to make the decision. We have the following considerations:

1. We can initially identify duplicates based on fields of high accuracy and high selectivity: PMID and DOI (Digital Object Identifier).
2. We may partition the remaining set of records based on some field with full applicability and high accuracy. Considering the deduplication process as a natural join operation, we can adopt hash-joining optimization techniques here (see further). The only qualified field for our data set is YEAR.
3. Normalization of record fields is carried out as necessary. For example, page numbers may be represented as ‘112–8’ or ‘112–118’.
4. A combination of two non-decisive fields may uniquely identify a record; for example (ISSN or EISSN) + Journal-Title, Journal-Title + Title, Journal-Title + Paging-Info, etc. Each of them can be developed into a rule to judge if two records are actually the same.
5. For articles not deduplicated based on the aforementioned rules, we should next consider text attributes with lower accuracy (JOURNAL NAME, TITLE, PAGING GROUP and AUTHORS, Table 2).

In the following sections, we explain in detail the two most important and time-consuming challenges in the deduplication problem: (i) the complexity of join operation, and (ii) the approximate matching-based text comparison. We then (iii) discuss the estimation of the time needed to compare two records and, finally, (iv) present the detailed deduplication algorithm in pseudocode.

**Table 2.** Categorization of article fields

Category	Field-group	Notes
Decisive	PMID	Median/low applicability
		Single selectivity
		High accuracy
	DOI	Median/low applicability
		Single selectivity
		High accuracy
Reliable partially decisive	Year	High applicability (rarely null)
		Low selectivity
		High accuracy
	ISSN and EISSN	Median applicability
		Median selectivity
		High accuracy
	Journal name	High applicability
		Median selectivity
		Median accuracy
		Abbreviations are common
	Title	High Applicability
		High Selectivity
		Median Accuracy
		Missing parts case exists
Useful but not reliable	Paging group (volume and issue and page)	Median applicability
		High selectivity
		Median accuracy
		Missing parts are common
	Author list	High applicability
		High selectivity
		Low accuracy
		Missing some author is not rare
		Name word order may vary

Record fields can be categorized according to their applicability, selectivity and accuracy. Applicability is the number of non-null values/number of total records. If a field has very few null/empty values, it has high applicability. Selectivity: the average selectivity of a field is 1 – (1/number unique field values.) If a field value of the field is shared by only very few records, the field have high selectivity. Especially, if we say a field has single selectivity or decisive, it means that any non-null value of this field is unique among records. Accuracy is the average probability of correctness of any value in a field. If a field has low accuracy, it is not a good idea to use it as a duplication indicator.

### The complexity of join operation

To find duplicate records from two lists with N records in each list, a naïve join operation is to compare every record in one list with every record in the other list. This join requires N^2^ record comparisons to be performed. Metta employs five search engines and so five lists of results will be returned for each query. To perform deduplication for the five lists, multiple join operations are needed. If each search engine returns N records for a query, then the total number of record comparisons required depends on the duplicate rate among the records from these lists, with a higher duplicate rate leading to a lower number of comparisons. Generally, the number of comparison operations needed to perform deduplication is k × N^2^ in the worst case, where k is some constant (k = 10 when there are no duplicate records). Now suppose we can use YEAR to partition the records by publication date (DP field), then records of a certain year from one search engine only need to compare with records from other search engines of the same year. This is a special case of *blocking*, an important technique for improving the computational efficiency of record linkage algorithms (12, 16–18). In blocking, all records are assigned to a set of blocks, usually of small sizes, such that records assigned to different blocks will not refer to the same entity. Therefore, pairwise comparisons of records are only necessary for records within the same block.

### Approximate text matching comparison

The text comparison problem has two layers: first, to compare word sequences (sequence matching), and second, to compare if two words are the same (string comparison).

#### Sequence matching

For example, consider a case in which the two records are actually the same. To identify this, we have to do the following steps: normalization, prefix-based string comparison and a longest-common-subsequence (LCS) matching (19). This problem is similar to that tackled by acronym resolution algorithms (e.g. 20).

Example:

A. Journal Title = Journal of psychosomatic research

B. Journal Title = J-Psychosom-Res

After normalization:

A. Journal Title = [Journal, psychosomatic, research] (where stop word ‘of’ is ignored)

B. Journal Title = [J, Psychosom, Res]

Then prefix-based string comparison:

J matches Journal, Psychosom matches psychosomatic, Res matches research

Finally the LCS matching algorithm:

We use the following formula to estimate the similarity between the two values:

Similarity (A, B) = LCS(A, B)/MIN_LEN(A, B)

Here LCS(A, B) = 3, LEN(A) = LEN(B) = 3, so the similarity is 100%.

The well-known best solution for LCS problem costs O(L_1_ × L_2_) time, where L_1_ and L_2_ are the lengths of two sequences, respectively. (Here the length of a sequence is the number of words in it.)

We define following formula (1) for approximation-based text comparison and we require that the similarity must be higher than 0.8 for two sequences to be considered matched.
(1)
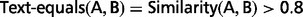

The choice of 0.8 as threshold was determined empirically, as it leads to optimal deduplication without introducing errors (see later).

#### String comparison

String comparison is not a trivial problem, as different records could use different encoding schemes (e.g. ‘Hello’ versus ‘Hḗllo’). Performing character-by-character comparison of Hello versus Hḗllo, again using the LCS algorithm, there are four matching characters out of five. So the similarity is 80%, and the cost of time is O(W_1_ × W_2_), where W_1_, W_2_ are the word lengths.

### Overall estimation of the time needed to compare two records

The computational cost for approximate matching text comparison can be estimated by:
(2)
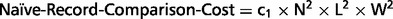

where c_1_ is some constant, N is the average number of records for a query, L is the average word sequence length (in journal name, title or author list) and W is the average word length. In a typical case with N = 300, L = 10 and W = 8, the cost is 576 000 000 even when c_1_ = 1. This would take tens of seconds to finish. If there is no need to perform approximate matching text comparison (e.g. when record matches can be quickly determined using fields like DOI and PMID), the cost would be:
(3)
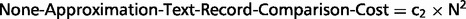

where c_2_ is some constant usually much smaller than c_1_ × L^2 ^× W^2^. When the number of records (N) is not large, the cost is acceptably small.

If we perform blocking on YEAR and implement a hash-join mechanism to put the results together, the cost could be further reduced to:
(4)
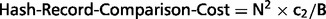

where B is the number of unique values of the hashing field.

### The detailed deduplication algorithm

The detailed deduplication algorithm as deployed in Metta can now be described. Note that the algorithm operates not on the standard BibTex format (Table 1) but on a version that has been extended with a few extra fields to provide information, i.e. PMID, DOI, ISSN and EISSN. Also note that this is based on blocking by YEAR. In our experience, only about 0.1% of records have missing YEAR field attributes; we handle them separately at the end of the algorithm. There are documented cases in which a database contains the wrong YEAR field for an article (ref. 15 and see later), but because considering this rare case would greatly increase the time needed, our algorithm does not attempt to deduplicate articles whose records do not match on YEAR.

### Metta-deduplication-algorithm

***f******unction Metta-******deduplication****:*

*# records partitioned by year & search-engine*

*input: Map<year, Map<search-engine, List<record>>> data_map*

*# records with year info missing*

*input: List<record> missing_year_records*

*output: List<Pair<record, record>> duplicate_pairs*

*duplicate_pairs = []*

*all_records = []*

*for each year in data_map:*

  *records_of_year = data_map[year]**[“**pubmed**”]*

  *for each search-engine in data_map[year]:*

    *if search-engine is**“**pubmed**” **continue;*

    *for each record A in data_map[year][search-engine]:*

      *for each record B in records_of_year:*

        *if Records-are-duplicate-based-on-Rules(A, B):*

          *duplicate_pairs.add(Pair<A, B>)*

        *else:*

          *records_of_year.add(A)*

  *all_records.addAll(records_of_year)*

*for each record C in missing_year_records:*

  *for each record D in all-records:*

    *if Records-are-Duplicate-based-on-Rule(C, D):*

      *duplicate_pairs.add(Pair<C, D>)*

    *else:*

      *all_records.add(C)*

*return duplicate_pairs*

### The rule-based record comparison algorithm

***f******unction****Records-are-duplicate-based-on-Rules:*

*input: record A*

*input: record B*

*output: bool*

*# quick denial rules*

*# rule 1*

*if A.PMID <> null and B.PMID <> null and A.PMID <> B.PMID:*

  *return false*

*# rule 2*

*if A.DOI <> null and B.DOI <> null and A.DOI <> B.DOI:*

  *return false*

*# rule 3*

*if A.ISSN <> null and B.ISSN <> null and A.ISSN <> B.ISSN:*

  *return false*

*if A.EISSN <> null and B.EISSN <> null and A.EISSN <> B.EISSN:*

*return false*

*# matching rules*

*# rule 4*

*if A.PMID == B.PMID: return true*

*# rule 5*

*if A.DOI == B.DOI: return true*

*# rule 6*

*if JournalEquals (A, B) and PageEquals (A, B): return true*

*# rule 7*

*if (JournalEquals (A, B) and TitleEquals (A, B)**\*

  *and AuthorEquals (A, B) and A.page = B.page): return true*

*# return false if no rule is matched*

*return false*

Note that, among the aforementioned seven rules, rules 1, 2 and 3 are denial rules that decide when two records do not correspond to the same article. The denial rules run quickly as the fields PMID, DOI, ISSN and EISSN are all easy to compare with, and there is a very high probability that two arbitrary records are not matched. The approximate matching rules usually take much more time to execute, so are implemented only when the earlier rules do not suffice to make a decision.

## Results and discussion

The rule-based algorithm was developed after examination of article record duplicates in Metta exports, and considered all of the cases described in the detailed analysis of duplicate publications carried out by Qi *et al.* (15). It uses a YEAR-based hash-join algorithm for blocking, followed by denial rules (DENIAL) to improve time efficiency and, finally, an approximate matching-based text comparison technique (APPROX) to find more potential matching record pairs. We evaluated the performance (execution time and accuracy) of four different approaches: (i) PLAIN, which uses blocking only; (ii) APPROX, which uses only approximation-based text matching but not denial rules; (iii) DENIAL, which uses only denial rules but not approximation-based text matching and (iv) APPROX + DENIAL, which uses both approximation-based text matching and denial rules (and is deployed for Metta).

We randomly selected 6265 records from the articles retrieved from queries entered by online users of Metta. Table 3 shows the processing times and the numbers of duplicate records found by different algorithms. The PLAIN approach found 727 duplicate pairs of articles in 4.1 s, whereas the most aggressive algorithm (APPROX) found 819 duplicate pairs (i.e. an additional 92 records) in 92 s. The best result was obtained for APPROX + DENIAL, which found the same 819 duplicates in only 32 s. By comparing different text approximation thresholds (0.7 versus 0.8 versus 0.9), we verified that 0.8 was the optimal setting insofar as it identified the most duplicate pairs (when the threshold was at 0.7, the algorithm made additional duplicate assignments, but these were errors).

**Table 3.** Performance of different algorithmic approaches

Approach	Time (s)	Duplicate records found
PLAIN	4.1	727
APPROX	92	819
DENIAL	3.2	727
APPROX + DENIAL	32	819

One of us (N.R.S.) manually verified that all of the 92 identified pairs (that required use of approximate matching techniques) were actual duplicate records. As reported previously (15), these duplicates were nearly all PubMed–EMBASE pairings, and they covered a wide range of situations: for example one database might put a supplement designation into the journal field, whereas another put it into the volume field. For articles published in online-only journals, sometimes article numbers were listed as page numbers and sometimes erroneous page numbers were entered. Different databases might give entirely different page numbers (e.g. 731–736 versus 730–735, or 41–42 versus e100044). We also observed a few cases in which article titles were written differently and a case where one database failed to include one author within the AUTHOR field. There was even one case in which the same article was indexed twice within EMBASE itself, and both articles were flagged as duplicates with the same PubMed article.

Note that the deduplication module of Metta is complementary to the deduplication module within NCBI (NCBI Batch Citation Matcher http://www.ncbi.nlm.nih.gov/pubmed/batchcitmatch), as Metta only deduplicates articles retrieved across databases, whereas the Batch Citation Matcher only deduplicates articles within PubMed itself. EndNote, a popular reference manager program, has a deduplication module that detects duplicates across databases, and so we compared its performance on the same set of 6265 records discussed earlier. Metta found 29 pairs of duplicate articles missed by EndNote (10 of these were detected using text approximation; 19 because they shared PMIDs). Conversely, EndNote found seven duplicate pairs (six of which were mismatches on publication year). EndNote also made 14 erroneous assignments of ‘duplicates’ that were not true duplicates; most of these were conference abstracts that were falsely identified with full-length publications that shared the same title.

As currently deployed as a module within the Metta metasearch engine, deduplication occurs during the process of exporting retrieved records to the user in the form of text files (displayed in either BibTeX or XML format). The export time includes the time needed for crawling records from individual search engines (it may vary based on the individual search engines’ responses) and the time needed to perform deduplication. In most cases, given a query with <3000 result records, the EXPORT would return deduplicated records within 2 min.

As stated in the Introduction, our goal is not to maximize the detection of all duplicate publications, but rather to minimize the loss of any distinct publications. In the current design, we consider the text matching problem as falling into the longest common sequence matching category. This design assumption is quite general, and can possibly be refined further when being applied to certain fields like AUTHORS, which generally agree in both the number and order of authors (11).

We have prepared a stand-alone script, written in Java, that allows anyone to deploy our deduplication module, starting with a set of article records in BibTeX format extended to include DOI, PMID, ISSN, EISSN, JOURNAL, TITLE, VOLUME, ISSUE, PAGE, AUTHOR and YEAR fields. The documentation and code are attached here as supplementary data, and are being released under the terms of GNU General Public License (version 3).

## Supplementary Data

Supplementary data are available at *Database* Online.
